# Enteroaggregative *Escherichia coli* Serotype O126:H27, Israel

**DOI:** 10.3201/eid0909.020695

**Published:** 2003-09

**Authors:** Gila Shazberg, Moshe Wolk, Herbert Schmidt, Iancu Sechter, Giora Gottesman, Dan Miron

**Affiliations:** * Bikur Cholim Hospital, Jerusalem, Israel; † Ministry of Health Central Laboratories, Jerusalem, Israel; ‡ Carl Gustav Carus Technische Universitaet, Dresden, Germany; § Meir Medical Center, Kfar-Saba, Israel; ¶ Haemek Medical Center, Afula, Israel; 1 Drs. Shazberg and Wolk contributed equally to this paper.

**Keywords:** Enteroaggregative *E. coli*, Ec serotypes, pCVD432, EcO126:H7, phage sensitivity

## Abstract

Enteroaggregative *Escherichia coli* (EAEC) is a newly diarrheagenic agent wherein several predominant serotypes are reported. We studied the association between those serotypes, as clonal indicators, and the trait of enteroaggregative adherence to host cells, tested by polymerase chain reaction. We also evaluated the clinical manifestations of infection in 17 hospitalized children by our most common EAEC serotype, O126:H27.

Enteroaggregative *Escherichia coli* (EAEC) is an emerging pathogen that causes diarrhea in many parts of the world, including children from Israel (*1*) and Jordan (*2*). This group of *E. coli* was preliminarily defined by its aggregative pattern of adherence (AA) to HEp-2 cells (*3*). Their identification was facilitated by a DNA probe developed from a plasmid (pCVC432 syn. pAA) necessary for expressing the aggregative phenotype (*4*). Based on that probe, a polymerase chain reaction (PCR) test was developed for screening EAEC strains (*5*). This test, which we used in this research, and another test using the same DNA probe (*6*), have been better indicators of diarrheagenic strains than the phenotypic Hep-2 cells test.

EAEC is a divergent group in terms of the organisms’ ability to induce diarrhea (*7*), the factors involved in attachment to host cells (*8*), and kinds of serotypes (*8*,*9*). Since certain EAEC serotypes were already prevalent throughout the world, we studied whether those strains could be found in our isolates of diarrheagenic EAEC. To simplify the detection of EAEC, we selected a bacteriophage active specifically on our clinically evaluated EAEC strains of *E. coli* O126:H27. EAEC have been rarely evaluated clinically in Israel. Here we address that problem by reporting clinical and microbiologic findings of children hospitalized with gastroenteritis in which our most common EAEC serotype, O126:H27, was found.

## The Study

Clinical signs and the laboratory findings were evaluated for 17 children <2 years of age, hospitalized in four pediatric wards in different areas of Israel. All these children had gastroenteritis attributable to EAEC or enterotoxigenic *E. coli* (ETEC) of serotype O126:H27 (Table 1).

**Table 1:** Bacteriologic parameters and clinical signs of children with *Escherichia coli* 0126:H27^a^

No.	Age (mo)	EAEC-PCR	Phage sensitivity	ST	Diarrhea (days)	Dehydration	IV fluid	Vomit	Fever	Concomitant clinical findings
1	6	+	+	-	+ (12)	+	+	+	+ 39	
2	1.5	+	+	-	+ (40)	+	+	-	-	Malabsorption, prolonged diarrhea
3	5	+	+	-	+ (4)	+	+	+	+ 40	
4	2	+	+	-	+ (4)	+	+	+	-	
5	15	+	+	-	+ (2)	+	+	+	+ 40	
6	21	+	+	-	+ (4)	+	+	+	+ 39	Tonsillitis
7	18	+	+	-	+ (1)	-	-	-	-	
8	16	+	+	-	+(9)	+	+	-	+39	Otitis
9	11	+	+	-	+ (8)	+	+	-	+40	Tonsillitis
10	16	+	+	-	+ (9)	+	+	+	+40	
11	15	+	+	-	+ (7)	-	+	-	+39	UTI
12	1	+	+	+	+(2)	+	+	-	-	
13	15	+	+	+	+(6)	-	+	-	+40	Tonsillitis leukocytosis
14	1.5	+	NT	+	+ (5)	-	+	-	+38.7	Meningitis
15	6	+	NT	+	+(1)	+	+	+	+40	
16	1 week	-	-	+	+(6)	+	+	-	-	
17	9	-	-	+	+ (5)	+	+	+	+40	

^a^EAEC, enteroaggregative *Escherichia coli*; ST, heat-stable toxin; UTI, urinary tract infections; NT, not tested.

Serotyping was performed (*10*). To determine O-antigen, cultures were heated to 120°C for 1 h, then checked for agglutination with specific O-antisera at 50°C overnight. For determination of H-antigen, motile cultures were grown overnight in nutrient broth, treated with 0.5% formaldehyde, and investigated for agglutination with specific H-sera at 50°C for 2 h.

To detect EAEC, we used PCR primers specific for a short sequence of the plasmid pAA of EAEC, which is necessary for adherence. Analysis for the presence of pCVD432 sequences was performed at the Institute of Hygiene and Microbiology, the University of Wuerzburg, and the Institute of Medical Microbiology and Hygiene, Technical University of Dresden, Germany. Briefly, *E. coli* were isolates grown overnight on L-agar, and a single colony was suspended in 50 μL of phosphate-buffered saline (PBS). Amplification was carried out in a total volume of 50 μL containing each nucleotide triphosphate at 200 μm, 30 pmol of each primer, 5 μL of 10-fold concentrated AmpliTaq DNA polymerase synthesis buffer, 1.5 mM MgCL_2_, 2.5 U AmpliTaq DNA polymerase (Applied Biosystems Applera, Weiterstadt, Germany), and 5 μL of template Oligonucleotides pCVD432/start (5′-CTG GCG AAA GAC TGT ATC AT-3′) and pCVD432/stop (5′-AAT GTA TAG AAA TCC GCT GT-3′) were purchased from Sigma-ARK GmbH (Darmstadt, Germany) (*5*). The PCR protocol comprises 30 rounds of amplification, each consisting of 30 s at 94°C, 60 s at 52°C, and 60 s at 72°C. The first cycle was preceded by a denaturation step of 10 min at 94°C, and the last extension cycle was followed by a final extension step of 10 min at 72°C.

Enterotoxins were determined by the Asialoganglioside-GM_1_ enzyme-linked immunosorbent assay (GM_1_-ELISA) method using the direct plate cultures technique. Heat-labile toxin (LT) was determined by GM1-ELISA using monoclonal antibodies against LT (*11*). Heat-stable toxin (ST) was determined in parallel in the same cultures by an inhibition-GM_1_-ELISA that used monoclonal anti-ST (*12*). The test was performed in two 96-well polystyrene microplates A&B (Nunc A/S Roskilde, Denmark) and comprises several steps. The plates were coated with GM_1_ by adding 0.1 mL of 0.3 nmol GM_1_ (Sigma, Rehovot, Israel) in 0.1 M PBS, pH=7.2, to each well. After the plates incubated overnight at room temperature, they were washed three times with PBS, blocked with 0.1% bovine serum albumin (BSA) in PBS for 30 min at 37°C, and finally washed once with PBS. To each of the GM_1_-coated wells in plate A was added 0.2 mL LB broth, Lennox medium, adjusted to 45 μg lincomycin/mL and 2.5 mg glucose/L. From each bacterial isolate, five colonies grown on McConkey agar were transferred directly into five separate wells. The cultures were grown for 24 h at 37°C with moderate shaking. Plate B (without the bacterial cultures) was processed after step 1 in a different way to determine ST. Briefly, plate B was coated with ST-CTB (consisting of the B-subunit of cholera toxin conjugated to ST) by adding 0.1 mL of ST-CTB in 0.1% BSA–PBS to each well and incubation of the plate at room temperature for 60 min. Then the plate was washed three times with PBS. To each well in plate B, 0.05 mL of culture medium from plate A was added (presumed to contain ST); immediately thereafter, 0.05 mL of the monoclonal antibody against ST (anti-ST) was added, and the plate was gently shaken. The plate was incubated for 90 min at room temperature and then washed three times with 0.05% PBS-Tween. After the culture medium was disposed of, plate A was washed three times with PBS-Tween. To each well, 0.1 mL of monoclonal antibody against LT (anti-LT) in PBS-BSA-Tween was then added. The plate was incubated for 90 min at room temperature and then washed three times with PBS-Tween. To each well of plates A and B we added 0.1 mL of goat anti-mouse immunoglobulin G–horseradish perioxidase (Jackson Immuno-Research Laboratories, West Grove, Pennsylvania) in PBS-BSA-Tween. The plates were incubated for 90 min at room temperature and then washed three times with PBS-Tween. Substrate was prepared by dissolving 10 mg of ortophenylene diamine (Sigma) in 10 mL of 0.1 M sodium citrate buffer (pH=4.5) to which 4 μL of 30% H_2_O_2_was added. To each well in plates A and B, 0.1 mL of this substrate solution was added. After 20 min, the plates were read at 450 nm in a Micro ELISA Auto Reader spectrophotometer (Dynatech Inc., Alexandria, VA).

When the optical density (OD) decreased >50% as compared with the OD of anti-ST mixed with ST negative control culture, which run in parallel to the experimental wells, the result was considered ST positive. When the OD value at 450 was >0.100 above the background, the result was considered LT positive. Since serotype O126:H27 was prevalent in our EAEC cultures, we tried to isolate bacteriophages specific to the EAEC of this serotype from sewage water. Five unrelated strains of EAEC serotype O126:H27 were used. One milliliter of an early logarithmic broth culture of each strain was seeded in a bottle of 50-mL nutrient broth. After incubation of 3 h at 37°C, 5 mL of sewage water was added to each bottle. After a new incubation of 6 h, cultures were killed by adding 1 mL of chloroform, followed by intensive shaking. The next day the supernatant of each bottle was tested for activity on the respective strain. The isolated phages were then diluted and purified twice by single plaque isolation (*13*). The five phages were active on EAEC strains of serotype O126:H27.

From July 1999 to December 2001, we collected and characterized 1,368 isolates of diarrheagenic *E. coli.* Of these isolates, 88 (6.4%) belonged to one of the five most common EAEC serotypes, i.e., serotype O126:H27 (n=48), O111:H21 (n=16), O125 (n=11), O44:H18 (n=11), O?:H10 (n=2) (Table 2). The percentages of EAEC PCR–positive strains (Table 2) were as follows: 73% in *E. coli* O126:H27 and 75% in *E. coli* O111:H21. In *E. coli* O125, the percentage was approximately 50%, and in *E. coli* O44:H18, unlike reported elsewhere (*14*), this percentage was low.

**Table 2:** Serotypes of enteroaggregative *Escherichia coli* (EAEC) evaluated by polymerase chain reaction

*E. coli* serotype	O126:H27	O111:H21	O125:Hx	O44:H18	O?:H10	Totals
No. positive	35 (73%)	12 (75%)	H9=5 H49=1 6(54.5%)	2 (18%)	1	56
No. negative	13 (27%)	4 (25%)	H49=3 H6= 2 H(-)=1 5(45.5%)	9 (82%)	1	32
Totals	48	16	11	11	2	88

To determine if the isolated phages were specific for the enteroaggregative strains of serotype O126:H27, the five phages were tested by spot test at routine test dilution on our EAEC and non–EAEC cultures of this serotype. Four phages were active on both kinds of strains. Only phage no. 4 was active on 33 of the 34 EAEC cultures and on 1 of 12 non-EAEC cultures (Table 3). The sensitivity of this phage was 97%, and its specificity was 91%. This phage could therefore be used as an indicator for AA in this *E. coli* serotype.

**Table 3:** Phage sensitivity *of Escherichia coli* O126H27 compared to EAEC-PCR^a^

Phage sensitivity^b^	EAEC-PCR positive	EAEC-PCR negative	
Positive	33 isolates	1 isolate	
Negative	1 isolate	11 isolates	

^a^EAEC PCR, enteroaggregative *Escherichia coli* polymerase chain reaction. ^b^Sensitivity = 97%; specificity = 91%.

*E. coli* O126: H27 was found in stools from 17 children in four pediatric wards in various areas in Israel (Table 1). The stools were watery; no mucus or blood was seen. Most of the children were dehydrated and needed IV treatment with fluids and electrolytes. Some children vomited several times. All 17 patients had a normal leukocyte count for age. Twelve of them had high fever (38.7°C–40°C). Three of these 12 children had diarrhea concomitant with other diseases (patients 11, 13, and 14). Stool cultures of these three children were taken as part of an investigation of febrile disease. The same three children received antibiotic treatment; all others recovered without antibiotics. The length of hospitalization was 2–8 days. The duration of diarrhea was 1–40 days (median 5 days) starting, in some cases, before hospitalization. ST was produced in six patients (nos. 12–17), while LT was not produced in any. Five patients (nos. 1, 2, 8, 9, 10) had prolonged diarrhea of >1 week, characteristic of EAEC (*15*).

## Conclusions

In our patients, EAEC serotype O126:H27 appears to be a pathogenic agent of young children who require hospitalization and dehydration treatment. This same serotype has been reported as a common cause of diarrhea in children from England (*16*), Japan (*17*), and Bangladesh (*9*). However, we were not able to associate that serotype exclusively with the enteroaggregative pathotype, since nonaggregative Ec O126;H27 strains from hospitalized children (patients 16 and 17 in Table 1) produced ST and might therefore belong to the pathotype of ETEC.

However, ST was apparently not the main diarrheagenic factor, since in the five children with prolonged diarrhea no ST was produced. Some other kind of toxin was probably involved in these cases. In strains from some patients (Table 1, numbers 12–15) we found both traits of EAEC and ETEC in the same organism. A simple test to identify EAEC in routine laboratory work is needed. A possible solution is to use a phage sensitivity test in addition to serotyping, such as we used here for EAEC O126:H27. Preliminary results suggest that this test (Table 3) is a reliable indicator. If this fact is confirmed on a large number of strains, specific phages might also be selected for EAEC of other serotypes.

The obvious accumulation of pCVD432-positive *E. coli* of serotype O126:H27 suggests that we found a clone that spread in Israel and probably has a selective advantage.
